# A new species of *Paracedicus* Fet, 1993 (Araneae, Desidae) from Turkey

**DOI:** 10.3897/BDJ.11.e109714

**Published:** 2023-08-17

**Authors:** Gökhan Gündüz

**Affiliations:** 1 Bursa Uludag University Graduate School of Natural and Applied Sciences, Zoology Section, Bursa, Turkiye Bursa Uludag University Graduate School of Natural and Applied Sciences, Zoology Section Bursa Turkiye

**Keywords:** Cedicinae, description, Eastern Anatolia, spider, Turkey

## Abstract

**Background:**

The desid spider genus *Paracedicus* Fet, 1993 comprises seven species distributed in the western Palaearctic. From Turkey, only *Paracedicusbaram* Levy, 2007 has been recorded so far.

**New information:**

A new species, *Paracedicusturcicus* sp. nov., is described, based on specimens of both sexes collected in Eastern Anatolia. Detailed morphological description and photographs are provided. Additionally, a key to all species of the genus and a distribution map are provided.

## Introduction

*Paracedicus* Fet, 1993 consists of seven species distributed in the western Palaearctic Region. Amongst these species; *P.feti* Marusik and Guseinov, 2003 is known from Azerbaijan; *P.ephthalitus* (Fet, 1993) from southern Turkmenistan; *P.gennadii* (Fet, 1993) from Iran and Turkmenistan. *P.kasatkini* Zamani and Marusik, 2017 and *P.darvishi* Mirshamsi, 2018 are distributed in northeast Iran. *P.baram* is known from Israel and Turkey; *P.geshur* Levy, 2007 from Israel (WSC 2023).

Studies on the genus are still not at a sufficient level. Discovery of new findings related to the genus is quite likely, especially in relatively less explored regions such as eastern and north-eastern Turkey.

In this study, a new species, *P.turcicus* sp. nov., is described, based on specimens collected from the Eastern Anatolian Region of Turkey. A key to all species of the genus and their distribution map are provided. In Turkey, the genus was recorded for the first time by Akpınar (2019) with *P.baram*. With this study, the number of the species of the genus is raised to two in Turkey.

## Materials and methods

All specimens were examined and measured using an Olympus SZ 11. Photographs were taken using a YW500 camera attached to the stereomicroscope. Measurements were obtained using S-EYE software from the dorsal side of the body parts and are reported in millimetres (mm). The lengths of leg segments are listed as follows: total length (femur, patella, tibia, metatarsus, tarsus). Prior to photography, the epigyne was cleared in a 10% potassium hydroxide (KOH) aqueous solution. The description and photographs of the male palp are based on the left one. The colouration pattern is described, based on specimens preserved in 70% ethanol. The morphological terminology follows Marusik and Guseinov (2003). The taxonomy and distribution follow the WSC (2023).

The following abbreviations are used in the text and figures:

- Eyes: **AER** ‒ anterior eye row, **ALE** ‒ anterior lateral eye, **AME** ‒ anterior median eye, **PER** ‒ posterior eye row, **PLE** ‒ posterior lateral eye, **PME** ‒ posterior median eye.
- Spination: **Do** ‒ dorsal, **Fe** ‒ femur, **Mt** ‒ metatarsus, **p** ‒ pair, **Pr** ‒ prolateral, **Pt** ‒ patella, **Re** ‒ retrolateral, **Ta** ‒ tarsus, **Ti** ‒ tibia, **Ve** ‒ ventral.
- Male palp: **c** – conductor, **e** – embolus, **eb** – embolic base, **ma** – median apophysis, **pa** – patellar apophysis, **t** – tegulum.
- Epigyne: **cd** – copulatory duct, **co** – copulatory opening, **ef** – epigastric furrow, **fd** – fertilisation duct, **fo** – fovea, **mp** – median plate, **om** – opaque membrane, **s** – spermatheca.
- Spinneret: **cl** – colulus.
- Chelicerae: **ctf** – cheliceral transverse folds.

**Depository**

**ZMUU** Zoological Museum of the Bursa Uludağ University, Turkey (R.S. Kaya).

## Taxon treatments

### Paracedicus turcicus sp. nov.

66E9E83D-9FE7-5D92-B33C-0844BBCE2EA4

50A0EAEA-00DA-491F-99B3-63736FF58B36

#### Materials

- **Type status:** Holotype. **Occurrence:** catalogNumber: G1; recordNumber: Paraturc2023_1; recordedBy: Gökhan Gündüz; individualCount: 1; sex: male; lifeStage: adult; preparations: 70% Ethanol Solution; occurrenceID: B59FEB68-115D-53BD-8D16-60822CCAF5ED; **Taxon:** scientificName: Paracedicusturcicus; **Location:** country: Turkey; stateProvince: Muş; locality: Korkut District, Konakdüzü Village; verbatimElevation: 1340 m; verbatimLatitude: 38°36'46.8"N; verbatimLongitude: 41°53'24.0"E; georeferenceProtocol: GPS; **Identification:** identifiedBy: Gökhan Gündüz; **Event:** samplingProtocol: Hand Aspirator; eventDate: 10/04/2018; **Record Level:** language: en
- **Type status:** Paratype. **Occurrence:** catalogNumber: G2; recordNumber: Paraturc2023_2; recordedBy: Gökhan Gündüz; individualCount: 1; sex: female; lifeStage: adult; preparations: 70% Ethanol Solution; occurrenceID: 25C34AAB-FA79-5E20-A568-3602B4DB0C65; **Taxon:** scientificName: Paracedicusturcicus; **Location:** country: Turkey; stateProvince: Muş; locality: Korkut District, Konakdüzü Village; verbatimElevation: 1340 m; verbatimLatitude: 38°36'46.8"N; verbatimLongitude: 41°53'24.0"E; georeferenceProtocol: GPS; **Identification:** identifiedBy: Gökhan Gündüz; **Event:** samplingProtocol: Hand Aspirator; eventDate: 10/04/2018; **Record Level:** language: en
- **Type status:** Other material. **Occurrence:** catalogNumber: G3; recordNumber: Paraturc2023_3; recordedBy: Gökhan Gündüz; individualCount: 1; sex: male; lifeStage: adult; preparations: 70% Ethanol Solution; occurrenceID: B13A8C45-5361-529F-9EF8-307F58BAC3E5; **Taxon:** scientificName: Paracedicusturcicus; **Location:** country: Turkey; stateProvince: Muş; locality: Korkut District, Konakdüzü Village; verbatimElevation: 1340 m; verbatimLatitude: 38°36'46.8"N; verbatimLongitude: 41°53'24.0"E; georeferenceProtocol: GPS; **Identification:** identifiedBy: Gökhan Gündüz; **Event:** samplingProtocol: Hand Aspirator; eventDate: 10/04/2018; **Record Level:** language: en
- **Type status:** Other material. **Occurrence:** catalogNumber: G4; recordNumber: Paraturc2023_4; recordedBy: Gökhan Gündüz; individualCount: 1; sex: female; lifeStage: adult; preparations: 70% Ethanol Solution; occurrenceID: C9E59716-C219-51EC-A20B-30A963E47292; **Taxon:** scientificName: Paracedicusturcicus; **Location:** country: Turkey; stateProvince: Muş; locality: Korkut District, Konakdüzü Village; verbatimElevation: 1340 m; verbatimLatitude: 38°36'46.8"N; verbatimLongitude: 41°53'24.0"E; georeferenceProtocol: GPS; **Identification:** identifiedBy: Gökhan Gündüz; **Event:** samplingProtocol: Hand Aspirator; eventDate: 10/04/2018; **Record Level:** language: en

#### Description

**Figure 1.**
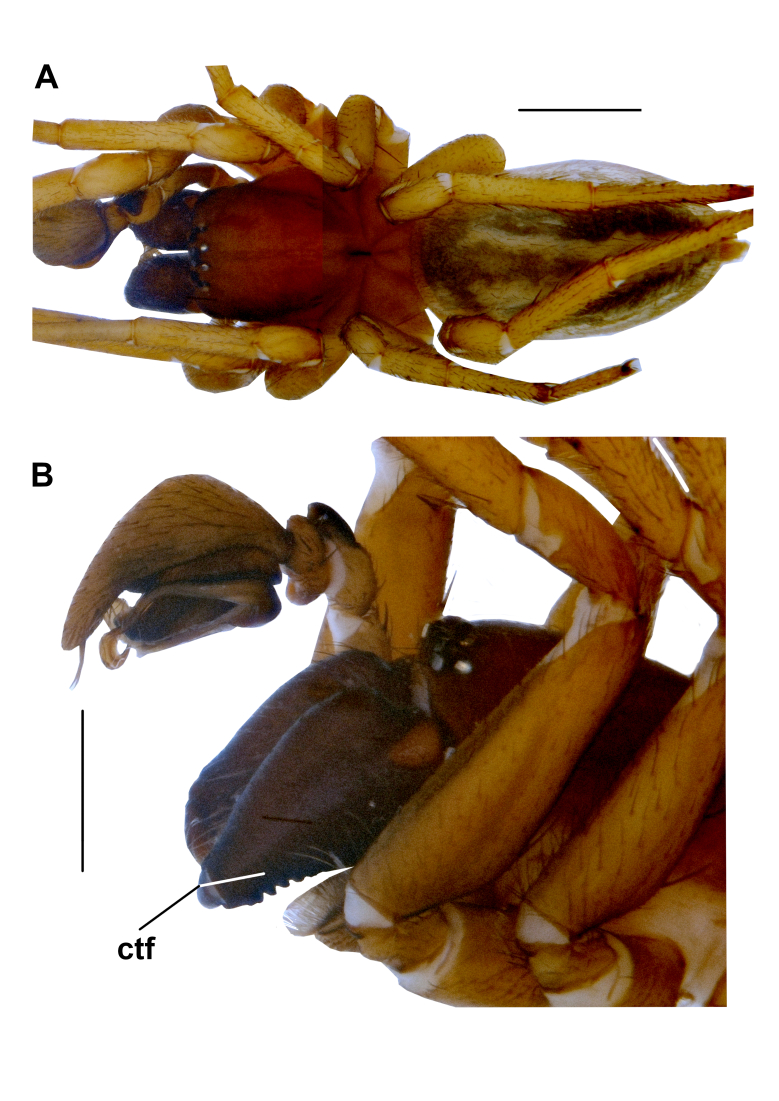
Male of *P.turcicus* sp. nov. **A** Habitus. **B** Anterior region of prosoma, lateral. Abbreviations: **ctf** – cheliceral transverse folds. Scale bars: 1 mm (A); 0.5 mm (B).

**Male: Holotype (ZMUU)**. Habitus as in Fig. 1A. Total body length: 5.83. Prosoma: Carapace length: 2.53, width: 1.73. Sternum length: 1.40, width: 1.06. Carapace brown (Fig. 1A). Sternum slightly reddish-yellow and droplet-shaped.

Legs: Legs yellow, with numerous spines (Fig. 1A). Measurements of legs: I: 6.47 (1.89, 0.75, 1.60, 1.31, 0.92), II: 5.64 (1.66, 0.64, 1.35, 1.14, 0.85), III: 4.83 (1.38, 0.58, 0.94, 1.18, 0.75), IV: 6.24 (1.86, 0.73, 1.49, 1.43, 0.73). Spination is given in Table 1.

Chelicerae: Chelicerae dark brown (Fig. 1B). Retromargin of cheliceral furrow with four gradually increasing teeth (the most distal one very small); promargin with five teeth, largest being fourth tooth distally.

Eye diameters and interdistances: AME: 0.07; ALE: 0.11; PME: 0.07; PLE: 0.08. ALE-AME: 0.10; AME-AME: 0.04; ALE-PLE: 0.04; PLE-PME: 0.15; PME-PME: 0.14; AME-PME: 0.05. PER: 0.75. AER: 0.58.

Gnathocoxae dark yellow with white patch distally, non-convergent. Labium similar in colour to gnathocoxae.

Abdomen: Abdomen greyish-yellow, with a dark pattern dorsally (Fig. 1A).

Spinnerets: Spinnerets light greyish-yellow. Apical segment approximately one-sixth length of basal segment.

**Figure 2.**
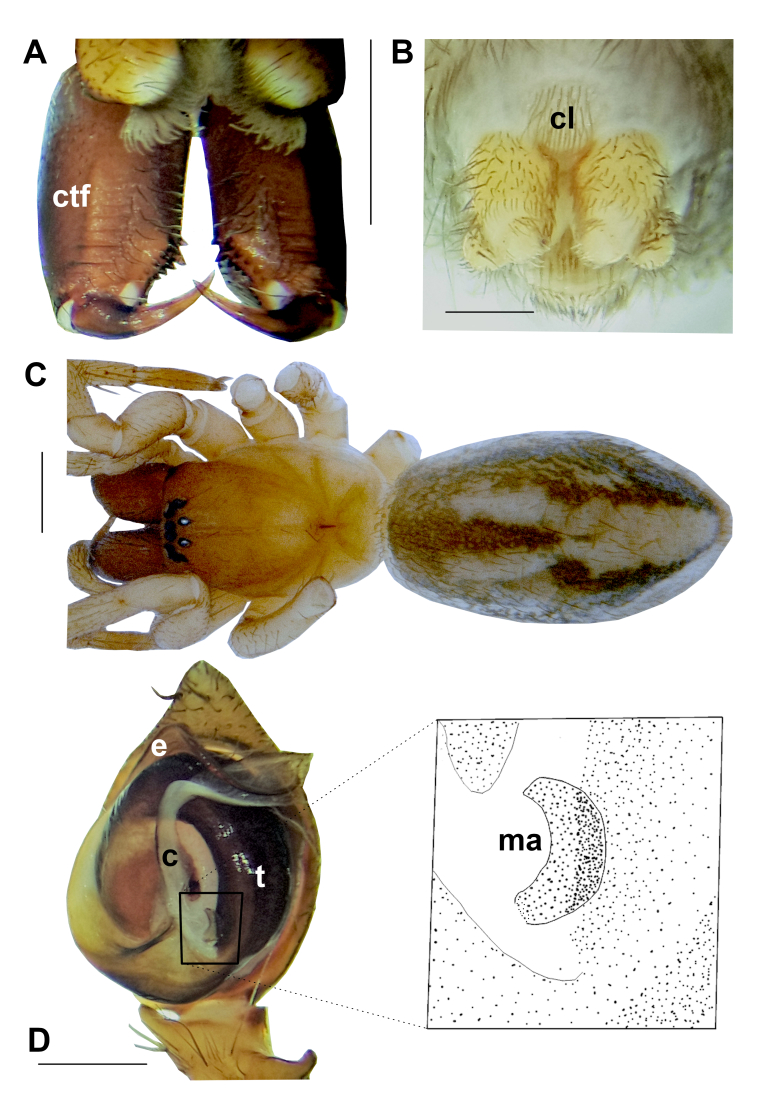
*P.turcicus* sp. nov. **A** Chelicerae of female, ventral. **B** Spinnerets of female, ventral. **C** Habitus, female. **D** Male palp, ventral. Abbreviations: **c** – conductor, **cl** – colulus, **ctf** – cheliceral transverse folds, **e** – embolus, **ma** – median apophysis, **t** – tegulum. Scale bars: 1.00 mm (A); 0.2 mm (B); 1 mm (C); 0.3 mm (D).

**Figure 3.**
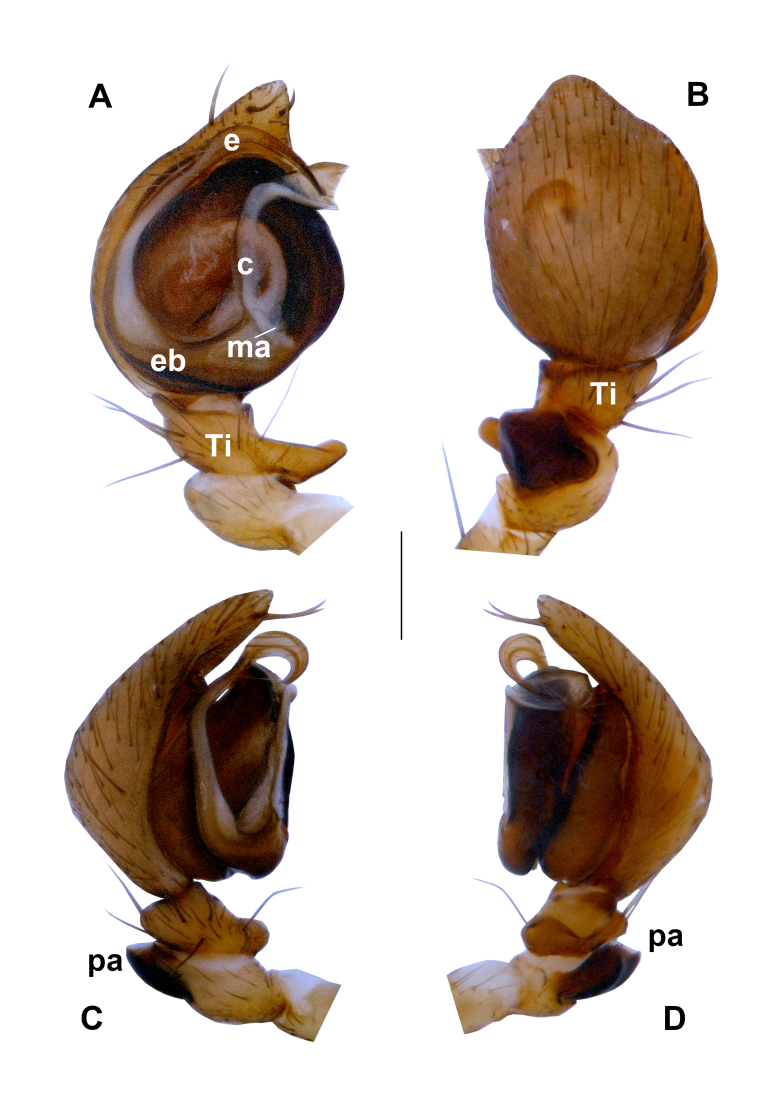
Male palp of *P.turcicus* sp. nov. **A** Ventral. **B** Dorsal. **C** Prolateral. **D** Retrolateral. Abbreviations: **c** – conductor, **e** – embolus, **eb** – embolic base, **ma** – median apophysis, **pa** – patellar apophysis, **Ti** ‒ tibia. Scale bar: 0.3 mm.

Palp: Femur without apophysis. Patella with two closely-positioned apophyses. One of them considerably larger (as wide as patella), with a blunt tip, dark-coloured, rectangular shape from dorsal view. Second one smaller, slightly bending retrolaterally and lighter. Tibia elongated diagonally and with several scattered long spines. Cymbium globular, with short hairs dorsally. Tegulum oval and without any projections or outgrowths. Median apophysis lamellar, semi-transparent, quite small and flat, crescent-shaped, adjacent to base of conductor and extending over dark-coloured tegulum (Fig. 2D). Conductor light, membranous, with its base located approximately in middle of tegulum, with truncated tip and bending retrolaterally. Embolic base thick. Embolus widening in middle part, narrowing towards to proximal, with sharp tip (Fig. 3).

**Female: Paratype (ZMUU)**. Habitus as in Fig. 2C. Total body length: 6.11. Prosoma: Carapace length: 2.66, width: 1.82. Sternum length: 1.53; width: 1.20. Carapace and sternum as in male, but lighter.

Legs: Legs as in male; but lighter. Measurements of legs: I: 5.35 (1.72, 0.63, 1.26, 1.01, 0.73), II: 4.52 (1.37, 0.56, 1.02, 0.93, 0.64), III: 4.29 (1.31, 0.47, 0.94, 0.88, 0.69), IV: 6.01 (1.76, 0.52, 1.52, 1.36, 0.85). Spination is given in Table 2.

Chelicerae: Chelicerae as in male, but lighter and with larger first (the most distal one) of retromarginal teeth (Fig. 2A).

Eye diameters and interdistances: AME: 0.09; ALE: 0.13; PME: 0.10; PLE: 0.09. ALE-AME: 0.07; AME-AME: 0.04; ALE-PLE: 0.03; PLE-PME: 0.13; PME-PME: 0.16; AME-PME: 0.05. PER: 0.84. AER: 0.65.

Gnathocoxae and labium as in male; but lighter.

Abdomen and spinnerets as in male; but slightly lighter (Fig. 2B, C).

**Figure 4.**
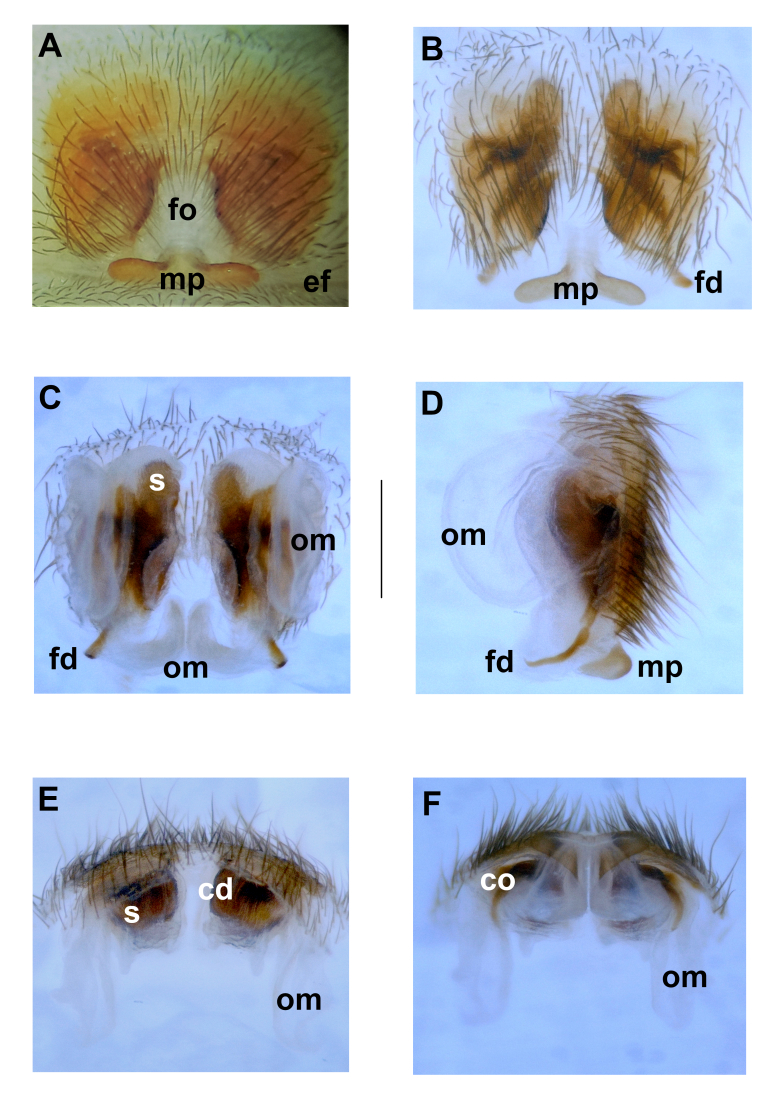
Epigyne and vulva of *P.turcicus* sp. nov. **A** Epigyne, ventral. **B** Macerated epigyne, ventral. **C** Vulva, dorsal. **D** Vulva, lateral. **E** Vulva, anterior. **F** Vulva, posterior. Abbreviations: **cd** – copulatory duct, **co** – copulatory opening, **ef** – epigastric furrow, **fd** – fertilisation duct, **fo** – fovea, **mp** – median plate, **om** – opaque membrane, **s** – spermatheca. Scale bar: 0.5 mm.

Epigyne: Simple, fovea surrounded by an intact sclerotised layer anteriorly and laterally. Fovea approximately twice as long as its width, narrowing towards anterior. Posteriorly, with extending transversely straight strip-shaped median plate tapering in middle and extending inwards. Vulva complex. Opaque and membranous structures extending inside epigyne, forming a pair of ear-like structures on both sides of vulva and covering copulatory ducts and spermathecae. Copulatory ducts wide. Spermathecae tubular, adjacent to ear-like opaque structures. Fertilisation ducts prominent and extending inwards (Fig. 4).

#### Diagnosis

**Paracedicus**: *Paracedicus* can be distinguished from related genera *Cedicus* Simon, 1875 and *Cedicoides* Charitonov, 1946 by genital characters. These can be listed as follows: median apophysis is present in species of *Paracedicus*, while it is absent in those of *Cedicus* and *Cedicoides*. In *Cedicoides* species, a prominent terminal apophysis is present in the palp, whereas this structure is absent in *Paracedicus* species. Additionally, in *Cedicoides* species, the conductor extends apically and the embolus is separated from the conductor, whereas in *Paracedicus*, the conductor is inclined retrolaterally and the embolus is situated within the conductor groove (Fet 1993, Marusik and Guseinov 2003, Levy 2007, Mirshamsi 2018).

The new species is most closely related to *P.feti*. It can be differentiated from *P.feti* and/or other congeners based on these characters: **1**- Size and shape of median apophysis (smaller in *P.feti*; larger in *P.baram*, *P.geshur* and *P.ephthalitus* than new species), **2**- thickness of embolus (thinner in *P.feti* than new species), **3**- orientation of conductor (not bending towards apical like *P.darvishi* or towards basal like *P.ephthalitus* and *P.gennadii* in new species), **4**- size and shape of patellar apophyses (The new species bears two apophyses; one being as wide as patella and rectangular, while the other is slender and inclined. Other congeners have less wide patellar apophyses.), **5**- morphology of palpal tibia (elongated diagonally in new species unlike *P.feti*), **6**- length of epigynal fovea, (longer than wide in new species, but wider than long in all other congeners, except for *P.kasatkini*), **7**- structure of vulva (covered with opaque membrane in new species unlike *P.feti*), **8**- colouration and pattern of abdomen (lighter than *P.darvishi* and *P.kasatkini* in new species; new species with an abdominal pattern unlike *P.kasatkini*).

#### Etymology

The specific name is an adjective referring to the country where it was found.

#### Distribution

**Figure 5.**
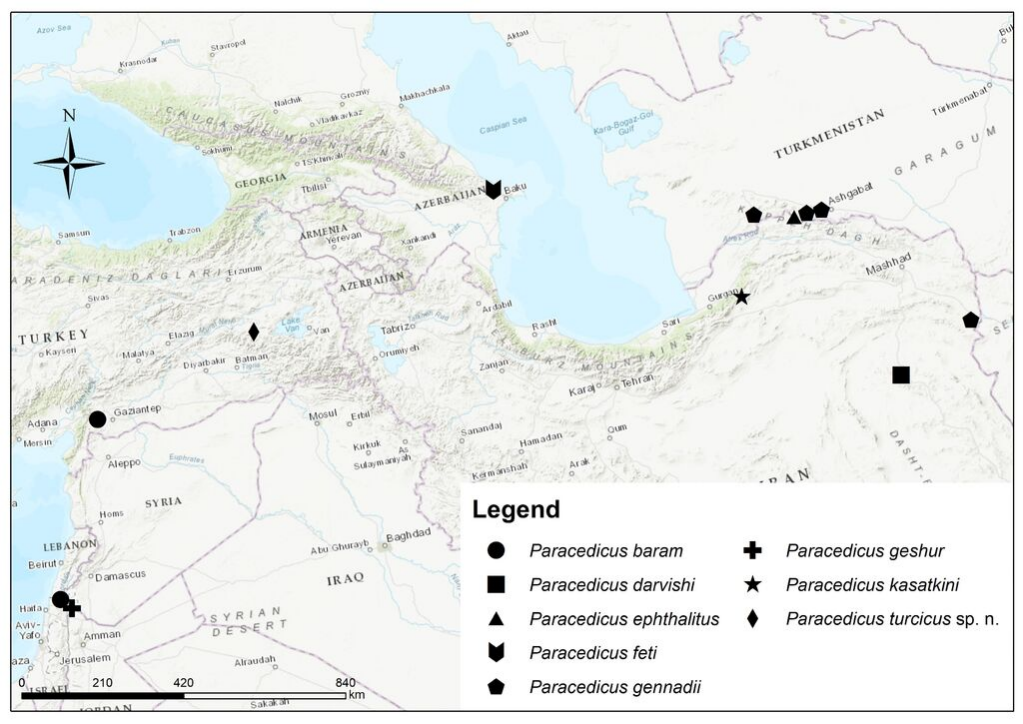
Distribution map of *Paracedicus* spp.

Known only from the type locality in Muş Province, eastern Turkey (Fig. 5).

#### Ecology

**Figure 6.**
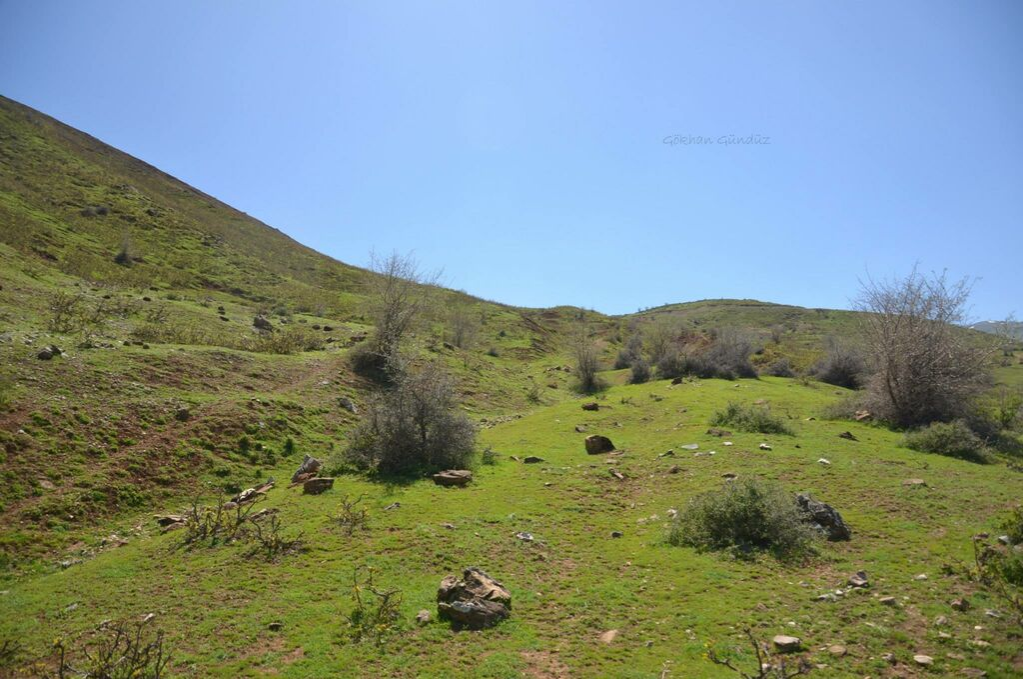
The type locality of *P.turcicus* sp. nov.; Muş Province, Korkut District, Konakdüzü Village.

The specimens were collected under stones from the northern slopes of mountains located in the southern part of Muş Basin, which is an important transit region between Central and Eastern Anatolia. The region is predominantly characterised by semi-arid oak forests and the presence of Irano-Turanian floral elements as ground vegetation. Furthermore, occurrences of maquis formations are observed in areas where oak forests have been degraded by anthropogenic impacts (Fig. 6).

#### Biology

The specimens were collected from their silken retreats under stones.

## Identification Keys

### Key to Species of *Paracedicus* spp.

**Table:** 

1	Male	2
–	Female	8
2	Both patellar apophyses less wide than patella and more or less pointed	3
–	One of patellar apophyses as wide as patella and blunt-ended, while other one smaller and pointed.	*P.turcicus* sp. nov.
3	Conductor bending towards basal	4
–	Conductor bending towards apical or towards retrolateral at almost a right angle	5
4	Median apophysis distinctly long and protruding	*P.ephthalitus*
–	Median apophysis not long and not protruding, tubercle-like-shaped	*P.gennadii*
5	Conductor bending towards apical	*P.darvishi*
–	Conductor bending towards retrolateral at almost a right angle	6
6	Tegular projection absent at proximal of conductor	*P.feti*
–	Tegular projection present at proximal of conductor	7
7	Median apophysis large and hook-shaped	*P.baram*
–	Median apophysis smaller and finger-like-shaped	*P.geshur*
8	Median plate absent	9
–	Median plate present	10
9	Fovea wider than long	*P.gennadii*
–	Fovea longer than wide	*P.kasatkini*
10	Epigyne with teeth	11
–	Epigyne without teeth	12
11	Median plate wider than fovea	*P.baram*
–	Median plate as wide as fovea	*P.geshur*
12	Fovea twice as long as its width	*P.turcicus* sp. nov.
–	Fovea wider than long	13
13	Epigynal plate oval	*P.feti*
–	Epigynal plate more or less rectangular-shaped longer than wide (approximately 1.25 times)	*P.ephthalitus*

## Discussion

*Cedicus* was described in the family Agelenidae C. L. Koch, 1837 by Simon (1875). Lehtinen (1967) transferred it to the subfamily *Desinae* Pocock, 1895. However, Levy (1996) moved it back to Cybaeidae Banks, 1892. Due to several genital character inconsistencies with cybaeids, especially those of males, Marusik and Guseinov (2003) have proposed to retransfer of *Cedicus* and closely-related genera (*Paracedicus* and *Cedicoides*) to Desidae. These three genera were transferred to Desidae by Zamani and Marusik (2017). Then Marusik et al. (2023) placed *Paracedicus*, along with *Cedicus* and *Cedicoides*, within the subfamily *Cedicinae* Marusik, Zonstein & Koponen, 2023 in Desidae. Zamani and Marusik (2017) provides a comprehensive summary of the taxonomic history of the group.

When comparing the characteristics of the observed features in the new species with the data available in the literature, several noteworthy points can be listed, which could potentially be considered as important: within all *Paracedicus* species, the presence of median apophysis is quite evident. Amongst the sclerites of the palp, the structure that exhibits the greatest variation within the genus is the tegular projection, which is observed in the Israeli species, but not in others. Regarding the evaluation of patellar apophyses, the new species shows closer resemblance to the Israeli species in terms of its relatively blunt structure, size and lack of patellar teeth. Amongst all congeners, the largest patellar apophysis is observed in *P.turcicus* sp. nov. In *P.feti* and other species, the patellar apophyses are characterised by two sharp-pointed and relatively slender structures. Setting aside the structure of the patellar apophyses, *P.turcicus* sp. nov. appears to be most closely related to *P.feti* in terms of its overall resemblance.

Considering the preferred habitats of *Paracedicus* species and distribution of the genus, this study has provided additional evidence supporting the potential presence of other species in the region.

## Supplementary Material

XML Treatment for Paracedicus turcicus

## Figures and Tables

**Table 1.** Leg spination in male of *P.turcicus* sp. nov. (Do-Pr-Re-Ve, 7 = 3p+1, p: pair).

Legs	Fe	Pt	Ti	Mt	Ta
I	2-1-0-0	0-0-0-0	0-1-0-6	0-0-0-7	0-0-0-0
II	2-1-0-0	0-0-0-0	0-1-0-6	0-1-0-7	0-0-0-0
III	2-0-1-0	0-1-0-0	0-0-2-6	6-1-0-7	0-2-2-4
IV	2-0-1-0	0-0-0-0	0-2-2-6	6-1-0-7	0-2-2-4

**Table 2.** Leg spination in female of *P.turcicus* sp. nov. (Do-Pr-Re-Ve, 7 = 3p+1, p: pair).

Legs	Fe	Pt	Ti	Mt	Ta
I	2-1-0-0	0-0-0-0	0-0-0-7	0-0-0-7	0-0-0-0
II	2-1-0-0	0-0-0-0	0-0-0-6	0-1-0-7	0-0-0-0
III	2-2-1-0	0-1-0-0	2-0-0-6	4-2-2-4	0-0-2-0
IV	2-0-0-0	0-0-0-0	0-0-2-4	2-1-2-7	0-0-2-0
